# Triploid Atlantic salmon are physiologically disadvantaged at larger body sizes

**DOI:** 10.1038/s41598-025-30342-5

**Published:** 2025-12-02

**Authors:** Malthe Hvas, André Morin, Tom J. Hansen

**Affiliations:** 1 Research Group of Animal Welfare, Institute of Marine Research, 5984 Matredal, Norway; 2 Sustainable Aquaculture Laboratory – Temperate and Tropical (SALTT), Deakin University, Geelong, Australia; 3 Reproduction and Developmental Biology, Institute of Marine Research, 5984, Matredal, Norway

**Keywords:** Aerobic scope, Animal welfare, Cell size, Critical thermal maximum, Gill histology, Hypoxia tolerance, Ecology, Ecology, Physiology, Zoology

## Abstract

Triploid Atlantic salmon are sterile and used in aquaculture to prevent escapees from breeding in the wild. Meanwhile, triploids suffer poor animal welfare in the latter marine growth phase. Previous experiments have mainly tested smaller fish, and physiological differences between triploids and diploids tended to be subtle or non-existing. We therefore hypothesized that triploidy first becomes a disadvantage at larger body sizes where scaling constraints become more magnified in triploids owing to them having larger cells with lower surface to volume ratios. We measured metabolic rates, stress responses, hypoxia tolerance, and critical thermal maximum in big (≈3 kg) triploid and diploid Atlantic salmon. Additionally, we assessed gill histology metrics. Big triploids had higher standard metabolic rates, lower aerobic scopes, and reduced tolerances to hypoxia and thermal stress. Oxygen extraction coefficients were overall lower in triploids, suggesting reduced efficiency in gill oxygen uptake. This was further supported by lower lamellar densities which indicate less gill surface area. In conclusion, big triploid Atlantic salmon were more vulnerable to environmental extremes driven by oxygen supply limitation and higher basal maintenance costs. This provides a mechanistic explanation for why triploids become prone to animal welfare issues in the latter growth phase of marine aquaculture.

## Introduction

A major sustainability concern of sea cage-based Atlantic salmon (*Salmo salar*) aquaculture is the occurrence of escaped farm fish interbreeding with salmon in the wild^1,2^. Cultured salmon have been selectively bred for desirable production traits over multiple generations since the 1970’s^3,4^, and trade-offs in the domestication process make cultured salmon less adapted to surviving in the wild^5–7^. Introgression of domesticated genotypes may therefore hurt wild salmon populations that already are under pressure from other anthropogenic activities^2,8,9^.

Introgression can be avoided by using sterile fish in aquaculture productions. Presently, the only reliable method to create sterile fish at a commercial scale is via the induction of triploidy – that is three complete sets of chromosomes as opposed to two sets in normal diploid fish^10^. A triploid fish group is created by pressurizing fertilized eggs to prevent the extrusion of the second polar body from the female gamete and thereby retaining two maternal chromosome sets along with one paternal set^10,11^. A consequence of being triploid is that cells become larger as they contain 50% more DNA. Meanwhile, relative organ sizes and body proportions remain roughly similar. A triploid fish will therefore comprise of fewer but larger cells relative to a diploid counterpart of the same size^12^.

Triploidy may cause physiological disadvantages leading to reduced health in aquaculture. In Norway, the world’s largest producer of Atlantic salmon, it has been documented that triploids suffer reduced animal welfare particularly in the latter part of the marine growth phase relative to diploids^13,14^. The Norwegian Food Safety Authority therefore imposed a temporary moratorium after 2023 on the use of triploid Atlantic salmon in sea cage-based aquaculture^15^. However, triploids are presently still being used in other salmon producing countries such as Canada and Australia.

Notable animal welfare issues when using triploid Atlantic salmon in commercial sea cages include higher mortalities, increased occurrences of wounds and ulcers, and generally being more susceptible to infectious diseases^13,14,16,17^. Reduced growth during the marine phase, higher occurrences of emaciated fish, and lower quality gradation at harvest also make triploid Atlantic salmon less attractive from an economic point of view^18–21^.

The underlying physiological implications of being triploid has been extensively studied in salmonids to help understand the potential benefits and challenges in aquaculture^10,22,23^. It can here be theorized that having cells with an extra set of chromosomes should lead to higher basal maintenance costs, resulting in elevated standard metabolic rates (SMR) when at rest. Although this effect may be offset by triploids consisting of fewer cells. Larger cells with lower surface to volume ratios should also limit exchange rate capacities of oxygen between cells and intracellular spaces, potentially restricting maximum metabolic rates (MMR) during strenuous activities or acute stress. A potentially higher standard and lower maximum metabolic rate would both contribute to a reduced aerobic scope for supporting any energetically costly activity. A reduced aerobic scope leads to higher vulnerability to environmental hypoxia, a prevailing issue in salmon sea cages^24,25^. Moreover, as energetic demands in ectothermic fish increase with temperature while oxygen solubility in water decreases, a higher basal maintenance cost and a reduced capacity for oxygen uptake should then result in a lower thermal tolerance, which is a concern as summer heatwaves are projected to get worse and more frequent in the future in salmon producing regions^26,27^.

When investigating these above mentioned theoretical predictions, empirical studies on triploid salmonid physiology have occasionally found support for them, but often also reported no or subtle effects depending on experimental context. For instance, reduced aerobic scope has been implied in triploid brook char (*Salvelinus fontinalis*) owing to an elevated SMR^28^, and in triploid chinook salmon (*Oncorhynchus tshawytscha*) owing to a reduced oxygen carrying capacity of the blood^29^. Meanwhile triploid Atlantic salmon had a lower aerobic scope at 10.5 °C although it was similar to diploid counterparts at 3 °C^30^. In contrast, other studies found similar critical swimming speeds^30–32^, as well as similar metabolic rates between triploid and diploid salmonids^33–35^, indicating that triploidy did not impose a substantial physiological disadvantage. With regards to environmental vulnerability, indicators of lower hypoxia tolerance primarily at elevated temperatures has been found in different triploid salmonid species, albeit effects tended to be subtle^36–38^. Additionally, Bowden et al.,^35^ reported negligible differences in thermal tolerance between triploid and diploid Atlantic salmon while Verhille et al.^39^ reported impaired tolerance to high temperatures in triploid rainbow trout (*Oncorhynchus mykiss*).

A convincing and consistent physiological explanation for differences between triploid and diploid salmonids has therefore not yet been demonstrated. However, laboratory experiments have primarily utilised smaller fish and typically in freshwater, although the prevailing animal welfare issues first tend to emerge when triploid Atlantic salmon become much larger during the latter marine sea cage production phase^13,14^. It would therefore be interesting to consider the theoretical implication of physiological scaling effects across body size between diploids and triploids.

Larger-sized fish are generally assumed to have a lower thermal optimum and a lower aerobic scope owing to geometrical scaling effects causing oxygen supply limitation^40–42^. Moreover, this consequently implies that fish species may become smaller as an adaptation to global warming^43–45^. From this perspective, a triploid fish can be considered an experimental model that encompass certain aspects of being a larger-bodied animal due to their larger cell sizes and lower surface to volume ratios^46^, factors that likely impose comparable geometrical scaling constraints on functionality. An example of this is the growth patterns of muscle cells, where fish generally rely less on hyperplasia and more on hypertrophy of cells as they grow larger, and in adult Atlantic salmon continued muscle growth relies solely on hypertrophy^47,48^. Interestingly, diploid Atlantic salmon have approximately one-third more muscle fibres per myotome owing to higher rates of fibre recruitment and lower rates of hypertrophic growth than triploid counterparts^49^ highlighting that triploids indeed may functionally resemble larger animals.

In zebrafish (*Danio rerio*), triploid models have been established to investigate fundamental effects of different cell and genome sizes (46). Triploid zebrafish larvae have been reported to perform better in colder conditions, while they perform worse at higher temperatures and show slightly worse hypoxia tolerance than diploid counterparts, indicating oxygen supply limitations in more challenging conditions^50,51^.

Impairments to physiological capacities and environmental tolerance limits in triploids can therefore be theorized to be similar to what will happen as a fish becomes larger. Furthermore, the magnitude of reduced robustness with increasing body size should then be greater in triploid relative to diploid salmon in aquaculture contexts. This would explain why the latter marine growth phase is when triploids are reported to struggle the most^13,14^. From an applied aquaculture perspective, it would be valuable to investigate whether the welfare issues of triploid Atlantic salmon in the final phase of sea cage production indeed are a consequence of size-dependent rate limitations that become exacerbated by larger cell sizes, making them more vulnerable to various stressors when compared to diploid counterparts. If so, this would also make larger-sized triploids more vulnerable to summer heatwaves and hypoxia events in the sea cage environment.

The purpose of this study was to measure key physiological capacities of larger-sized (≈ 3 kg) triploid Atlantic salmon as compared to diploid counterparts when acclimated to a mid-seawater temperature of 12 °C. First, we performed respirometry trials to measure metabolic rate traits and acute hypoxia tolerance. Then we performed critical thermal maximum (CT max) trials and assessed haematological parameters in fish subjected to this imposed thermal stress. Additionally, we did gill histology analyses on all the fish tested to potentially provide a morphological link to the physiological data.

We hypothesized that the larger-sized triploid Atlantic salmon would have a lower maximum metabolic rate and lower aerobic scopes compared to diploid counterparts of similar sizes, driven by larger cells with higher surface to volume ratios limiting physiological rates at the cellular level. A reduced capacity for oxygen uptake in triploids should also translate into a reduced hypoxia tolerance and a reduced oxygen extraction coefficient. Acute thermal tolerance was also hypothesized to be lower in triploids owing to larger-sized cells making it more difficult to maintain homeostasis. Overall, we hoped to demonstrate an obvious and more consistent difference in physiological capacities and environmental limits between larger-sized diploid and triploid Atlantic salmon when compared to past experiments on smaller-sized fish.

## Methods

### Fish husbandry

The fish used for these experiments were produced and reared on site at the Matre Research station, Institute of Marine Research, Norway. The diploid fish group was made from crossing 12 females and one normal male (XY). The triploid fish group was made from the same parents as the diploids, where eggs were fertilized with frozen milt and pressurized for 5 min at 655 bar 300 minC (second meiotic division) post-fertilization.

Each group was incubated in a single tray before being moved to single square, gray, covered, fiberglass tanks (1 × 1 × 0.43 m) supplied with filtered, UV-treated flow-through freshwater. When reaching approximately 40 g the fish were moved to larger tanks (1.5 × 1.5 × 0.7 m). The fish were then maintained in these tanks until approximate sizes of 800 g whereafter they were moved to larger tanks (3 m in diameter, 5.6 m^3^ in volume), where they were growing until sizes of 2–3 kg.

From the initial feeding until the end of the experiment, the two fish groups were fed to satiation daily with size appropriate pellets (Skretting) as the fish grew. Once the groups smoltified, the tank environments were kept at 22–25 ppt and 7–9 °C.

One month prior to starting the experimental trials, the diploid and triploid fish groups were transferred to a new circular holding tank (3 m in diameter, 6.3 m^3^ in volume) with ~ 50 individuals per tank. The first week following movement into the new tanks they were maintained at 25 ppt and 9 °C, corresponding to the previous recent tank environment. Thereafter the water quality parameters were changed to 12 °C and full strength seawater of 34 ppt which were the conditions used for the experimental trials.

In the final holding tanks, aerated, filtered, and UV-C treated water was supplied at a continuous flow-through of 130 l min^-1^ to provide normoxia (above 80% saturation at all times) and to remove waste products. Meanwhile, the fish were still being fed size appropriate commercial feed pellets (Skretting, Norway) in excess daily via automatic feeding devices and were subjected to a 12:12 photoperiod. The constant water temperature of 12 °C was maintained and controlled automatically via computer software (SDMatre, Normatic AS, Nordfjordeid, Norway) by mixing of ambient and heated water reservoirs in header tanks above the holding tanks. At the time of the experimental trials, the fish groups were approximately 2.5 years old.

The experimental trials were performed between September and November 2024 following relevant guidelines and regulations after having obtained approval from The Norwegian Food Safety Authorities (FOTS Id number 30720) for the use of animals in scientific research. Humane endpoints were defined by the endpoint of the experimental trials (See section "Respirometry setup and protocol" and "Thermal challenge setup and protocol"). Data from this study are reported in accordance with the ARRIVE guidelines.

### Respirometry setup and protocol

To measure oxygen uptake rates (MO_2_) in big diploid and triploid Atlantic salmon, an automatic static intermittent flow respirometry system was used (Loligo Systems, Denmark). The system consisted of three cylindrical shaped acrylic chambers submerged in their own separate water tanks so that three fish could be tested at the same time in parallel. The chambers were 125 cm long with a 30 cm internal diameter and were connected to plastic tubes forming an internal loop that passed through a flow-through oxygen sensor cell (measuring at 1 Hz) and a circulation pump. For intermittent flush periods, each chamber was also connected to an open loop with a flush pump (Eheim Universal 3400) with a flow capacity of 58 l min^-1^. A temperature probe along with the flush pumps and fibre cables to the oxygen sensors were connected to a computer running the AutoResp software (Loligo Systems). The oxygen sensor had been carefully calibrated according to the manufacturer’s instructions before experimental trials started. The rectangular water tanks containing a respirometry chamber and associated equipment were 200 × 60 × 50 cm, and each tank had their own flow-through water supply of ≈30 l min^-1^. The water supply was of the same quality as in the holding tank (12 °C, 34 ppt) and ensured a continuous exchange with clean aerated water of the correct temperature. To mitigate potential disturbances to the fish while in the chambers, the lights were dimmed and other activities in the laboratory hall were not allowed when trials were running.

Before each respirometry trial feed was withheld from a holding tank for one day to mitigate confounding metabolic effects associated with feeding and digestion to facilitate more accurate standard metabolic rate estimates^52^. The next day a fish was netted from a holding tank and transferred into a respirometry chamber. As the setup was located adjacent to the holding tanks, the duration of air-exposure and handling of the fish was brief and consistent between individuals (≈ 1 min). After having sealed off the respirometer and ensured that any air bubbles were removed, measurement cycles of MO_2_ were started. Automated intermittent-closed measurement cycles were then repeated over the next 24 h. The measurement cycles used were 10 min long and consisted of a 5-min closed measurement period followed by a 4.75-min flush period to reestablish oxygen levels and a 0.25-min closed wait period to stabilize flow conditions before repeating the cycle. An exception to this was made for the first few cycles at the start when MO_2_ was highest where a 4-min closed period was used instead.

After 24 h, the response to progressive hypoxia was assessed by omitting the flush period from the measurement cycle. When MO_2_ started to decrease below the baseline level established in normoxia and prior to cessation of active gill ventilation, the chambers were opened and the fish were removed and euthanized by immersion in an overdose of anaesthetics (300 mg l^−1^ Tricaine mesylate, Finquel vet.). The now empty chambers were then resealed to account for background respiration rates where a minimum of three cycles were completed in normoxia.

A blood sample was drawn via caudal puncture with a heparinized syringe to make duplicate blood smears per fish for later assessments of red blood cell sizes to confirm ploidy status. Then the weight and fork length of the fish were recorded. Additionally, the second right gill arch was removed, gently flushed with water to remove blood, and preserved in a 4% buffered formalin solution for 72 h whereafter it was transferred to a 70% ethanol solution and stored at 4 °C until later processing. Lastly, the fish were opened to assess maturation status. In the diploid fish the gonads were removed and weighed for calculation of the gonadosomatic index (GSI). The triploids all had poorly developed gonads, and we therefore did not bother to weigh them. Afterwards to prepare for more respirometry trials, the water tanks and equipment were cleaned. In total 15 diploid and 15 triploid Atlantic salmon were measured individually in the respirometry setup.

### Thermal challenge setup and protocol

To measure the critical thermal maximum (CT max) in larger-sized diploid and triploid Atlantic salmon, a modified holding tank of the same dimensions as used for maintaining the fish groups was used. A pool heater (Evolution 2 VFS Pool heater, 9 kW, 400 v, 13 A) was installed to heat and circulate water within the tank. The inflow of the circulating water was kept above the water surface to provide gas equilibration with the air which also served to avoid excessive super saturation of oxygen during heating, which was confirmed by an oxygen sensor within the tank. To ensure a controlled and steady increase in water temperature, the pool heater was connected to a relay box that could be switched on and off by computer software as directed by input of a thermal probe submerged within the tank (OmniCTRL, Loligo Systems, Denmark).

Prior to a thermal challenge, five fish were netted from a holding tank and transferred to the thermal trial tank. They were then allowed to acclimate in the trial tank overnight. During this time the trial tank received a normal open-flow water supply as in the holding tanks (12 °C, 34 ppt). The next day, the water supply was stopped, and the water height slowly reduced to ≈ 0.5 m and a water volume of ≈ 3.5 m^3^. The thermal challenge was then initiated and consisted of heating the water steadily by 3 °C per hour. Meanwhile the fish were carefully observed. When a fish eventually displayed a loss of equilibrium, it was immediately removed and knocked out with a blow to the head. Time and temperature were noted whereafter the fish was sampled in the same way as described for the respirometry protocol. Additionally, blood samples were here also used for haematological analyses where 1.5 ml blood from each fish were transferred to Eppendorf tubes and centrifuged at 6000 g for 5 min at 4 °C, whereafter the plasma was stored at stored at −80 °C for later analyses. In total 15 diploid and 15 triploid Atlantic salmon were tested in the thermal challenge setup.

### Confirmation of ploidy

To confirm ploidy status, blood smears from all fish tested were used to measure average red blood cell diameters with the assumptions that triploid fish have larger cells^53^. Blood smears were viewed with a Scion camera (CFW-1312 C) mounted on a DMRE light microscope (type 020–525.755). An image was taken for each blood smear at a resolution of 4.396 pixels μm^-1^ at 40 × magnification, and average cell diameter was calculated by automatically measuring hundreds of cells per picture using the ImageJ software.

### Haematological analyses

In the plasma samples obtained from fish subjected to the CT max trial, cortisol concentrations were measured with an ELISA assay kit in 20 µl subsamples (standard range: 10–800 ng ml^−1^, IBL International GmbH). Meanwhile, plasma osmolality was measured with freeze point determination in 20 µl subsamples with a Fiske 210 Micro-Sample Osmometer (Advanced Instruments). Additionally, concentrations of plasma glucose, lactate, Cl^-^, Na^+^, K^+^, and Ca^2+^ were measured in 65 µl subsamples using an ABL90 FLEX blood gas analyzer (Radiometer).

### Gill histology

Gill samples were passed through a benchtop histoprocessor (Leica TP 1020) according to the following protocol: 2 × 70%, 80%, 2 × 96%, and 2 × 100% ethanol, 2 × xylene, 2 × paraffin, and then embedded in blocks of paraffin and cut in 3 μm cross-sections with a rotary microtome (Thermo Fisher Microm HM 355 s). The sections were then mounted on glass slides and stained with haematoxylin–eosin-safranin according to standard protocols. Cross-sections were deparaffinised in xylene, rehydrated in a graded series of ethanol solutions, and rinsed with distilled water to complete the rehydration. Samples were immersed in haematoxylin for 1.5 min and then rinsed in distilled water for 4 min. Thereafter, samples were immersed in eosin for 1.5 min, dipped in distilled water, and treated with 96% ethanol for 45 s followed by 100% ethanol for 1 min. Samples were immersed in safranin for 10 s, treated with 100% ethanol for 1 min, and cleared in xylene for 10 min. Finally, coverslips were mounted on the slides and cross-sections were imaged using a digital slide scanner (Hamamatsu Photonics NanoZoomer S60).

Using NDP.view2 version 2.9.29 (Hamamatsu Photonics K. K., 2022) morphometric analysis were performed on 100 lamellae per fish. 5 gill filaments were randomly selected and 20 lamellae (10 × 2 lamellae) from each filament were chosen using stratified random sampling (2 proximal, 1 middle, and 2 distal regions). Lamellar density was quantified by measuring the distance spanned by 10 consecutive lamellae in each of the 5 regions, averaging these measurements, and extrapolating the density per mm. Furthermore, common gill health metrics were scored which included epithelial lifting, hyperplasia, clubbing, oedema, hyperemia, hypertrophy, lamellar fusion, aneurysm, and necrosis^54^. When no pathologies were present, lamellae were classified as being healthy. The interpretative approaches of Wolf et al.^55^ was adapted to ensure accurate histopathological diagnosis. This meant that only regions with lamellae that were full-length and symmetrical were scored, and tangentially sectioned regions or potential artifacts such as tissue aggregation or sloughing of cells were avoided. Lastly, all gill scoring was done blind to treatment groups to ensure objectivity.

### Calculations and data analysis

The MO_2_ (mg O_2_ kg^−1^ h^−1^) was calculated for each measurement period from the decreasing oxygen level over time:

$$MO_{2} = \frac{{\frac{{\Delta O_{2} }}{\Delta t}(V_{res} - V_{f} )}}{{W_{f} }}$$

ΔO2/Δt is here the change in mg O_2_ per hour, V_res_ is the volume of the respirometer, V_f_ is the volume of the fish, assuming a density of 1 kg l^−1^, and W_f_ is the weight of the fish.

The SMR was estimated as the mean of the 10% lowest values from the initial 24-h in the respirometer, after having removed any outliers exceeding ± 2 standard deviations of the mean^56^. The MMR was defined as the highest MO_2_ measured, coinciding with the start of the trial when the fish were maximally stressed owing to handling, air-exposure and subsequent confinement in the respirometer, a novel unfamiliar environment^57^. The absolute aerobic scope was expressed as the difference between MMR and SMR, while the factorial aerobic scope was expressed as MMR divided by SMR. The critical oxygen tension (P_crit_) was defined as the oxygen level before MO_2_ decreased below SMR as measured in normoxia^58,59^. The oxygen extraction coefficient (α) was expressed as MO_2_/PO_2_ at P_crit_ and MMR^60^.

The CT max was calculated as the set temperature reached plus the proportion of the time interval endured when a fish was removed, as the water was heating to and then stabilizing at the next set temperature.

Statistical differences between diploid and triploid Atlantic salmon in the various measured parameters were assessed with a t-test after having checked for equal variance and normal distribution of the data with Levene’s mean test and Shapiro-Wilks tests, respectively. If data did not adhere to these test assumptions, even after having performed a log transformation, a Welch’s t-test was used instead. Additionally, a two-way ANOVA with the Holm-Sidak post-hoc test was used to assess differences in α values between ploidies and MO_2_ traits as well as for gill morphology traits between ploidies and trial type. Lastly, linear regressions and Pearson’s correlation analyses were used to assess links between lamellar density and physiological performance metrics. A P-value below 0.05 was considered significant and data reported in the text are mean ± s.e.m. unless stated otherwise.

## Results

**Fig. 1:**
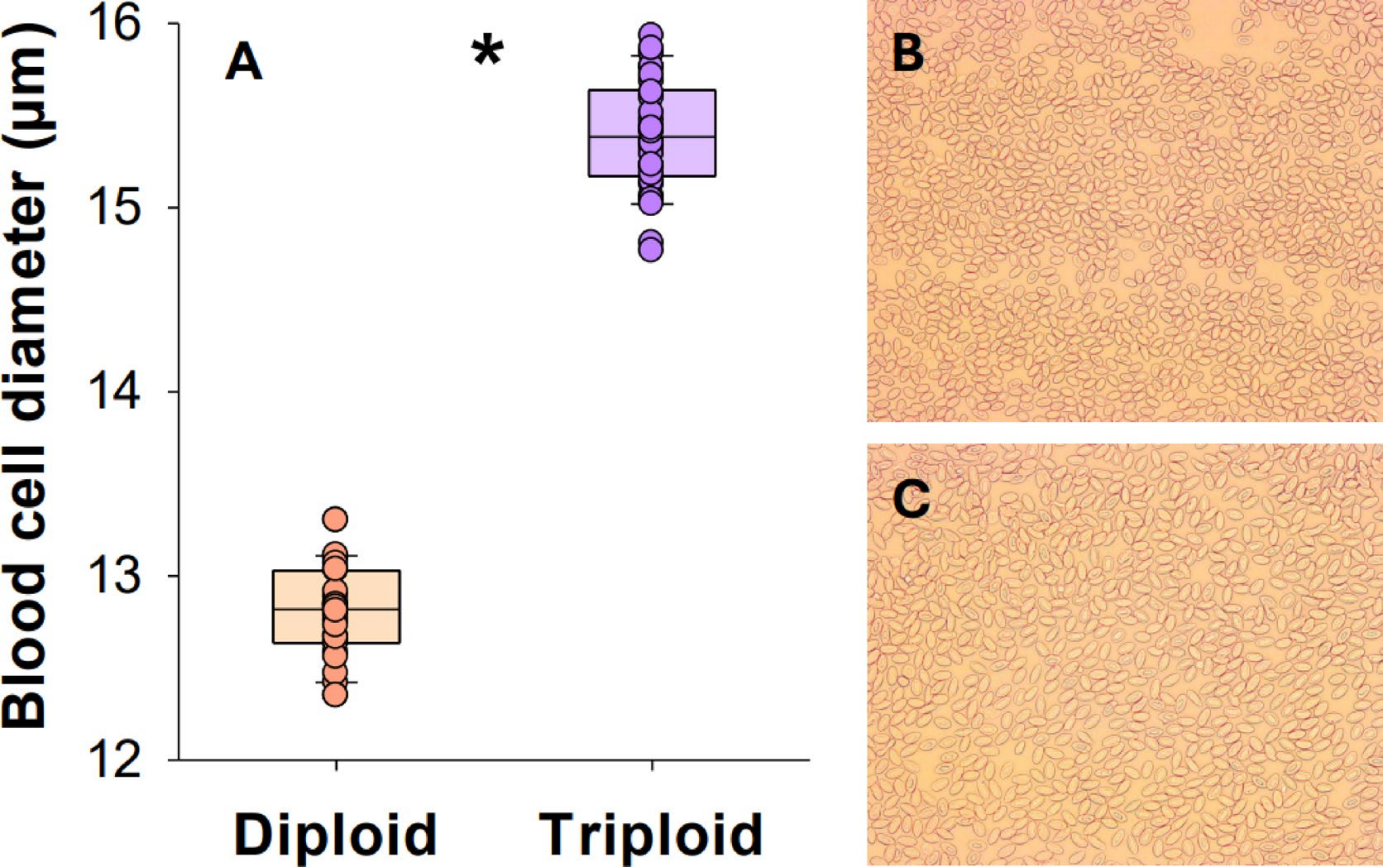
Diameter of blood cells in larger diploid and triploid Atlantic salmon (**A**). Data are shown as boxplots together with individual data points. The asterisk indicates a significant difference (t-test, *p* < 0.05) and n = 30. Additionally, representative photos of diploid (**B**) and triploid (**C**) red blood cells at 40 × magnification under a light microscope. Dimensions of both photos are 568 × 456 μm.

The correct ploidy status was confirmed in all tested individuals from size measurements of red blood cells. The mean diameter of red blood cells was 12.8 ± 0.04 µm in the diploids and 15.4 ± 0.06 µm in the triploids with no overlap between any individuals between ploidy group (t-test, t = −35.8, DF = 58, *P* < 0.0001) (Fig. 1A). As such, the sizes of diploid red blood cells appear noticeably smaller than in the triploids when viewed under a microscope (Fig. 1B and C).

The weight, length and condition factor were overall similar between the experimental groups in each trial type (Table 1). The only exception was that the diploids had a higher condition factor than the triploids tested in the CT max trials (t-test, t = 2.236, DF = 28, *P* = 0.0335). All of the fish tested were still immature. Across trial type the GSI (% body weight) in the diploids was 1.37 ± 0.20 in females and 0.17 ± 0.02 In males with a ratio of 1.3 females per male of the fish tested, and none of the triploids had developed gonads.

**Table 1:** Summary of size parameters in the experimental groups.

Trial	Ploidy	Weight (g)	Length (cm)	Condition factor
Respirometry	Diploid	2722 ± 125	59.0 ± 0.9	1.31 ± 0.02
	Triploid	2893 ± 105	60.8 ± 0.7	1.28 ± 0.02
CT max	Diploid	2690 ± 115	59.2 ± 0.9	1.29 ± 0.02^a^
	Triploid	2689 ± 110	60.1 ± 0.9	1.23 ± 0.02^b^

A statistical difference within trial type is indicated with different superscript letters (t-test, *p* < 0.05). Data are mean ± s.e.m and n = 15.

**Fig. 2:**
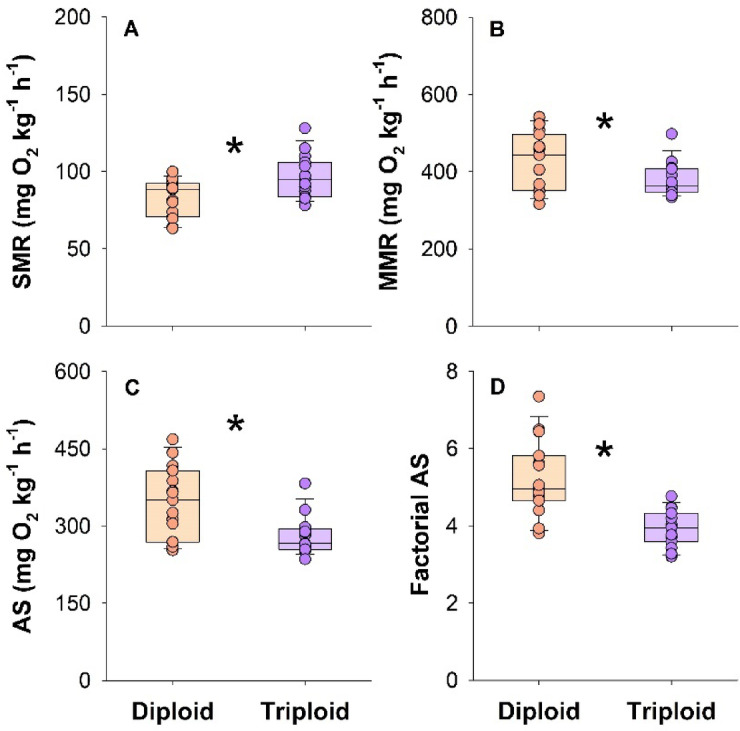
Metabolic rate traits in larger diploid and triploid Atlantic salmon. Standard metabolic rate (SMR) (**A**), maximum metabolic rate (MMR) (**B**), aerobic scope (AS) (**C**), and factorial AS (**D**). Data shown are boxplots together with individual data points. A significant difference is indicated with an asterisk (t-test, *P* < 0.05) (n = 15).

The diploids had a significantly lower SMR of 82.9 ± 3.1 compared to 97.0 ± 3.6 mg O_2_ kg^−1^ h^−1^ in the triploids (t-test, t = -2.969, DF = 28, *P* = 0.006) (Fig. 2A). The MMR was significantly higher in diploids at 428 ± 19 mg O_2_ kg^−1^ h^−1^ compared to 377 ± 10 mg O_2_ kg^−1^ h^−1^ in triploids (Welch’s t-test, t = 2.150, DF = 22.515, *P* = 0.043) (Fig. 2B). Both the resultant absolute and factorial aerobic scopes were significantly lower in the triploids compared to the diploids (Welch’s t-test, t = 3.208, DF = 21.354, *P* = 0.004 and logged t-test, t = 4.942, DF = 28, *P* < 0.001) (Fig. 2C and D).

**Fig. 3:**
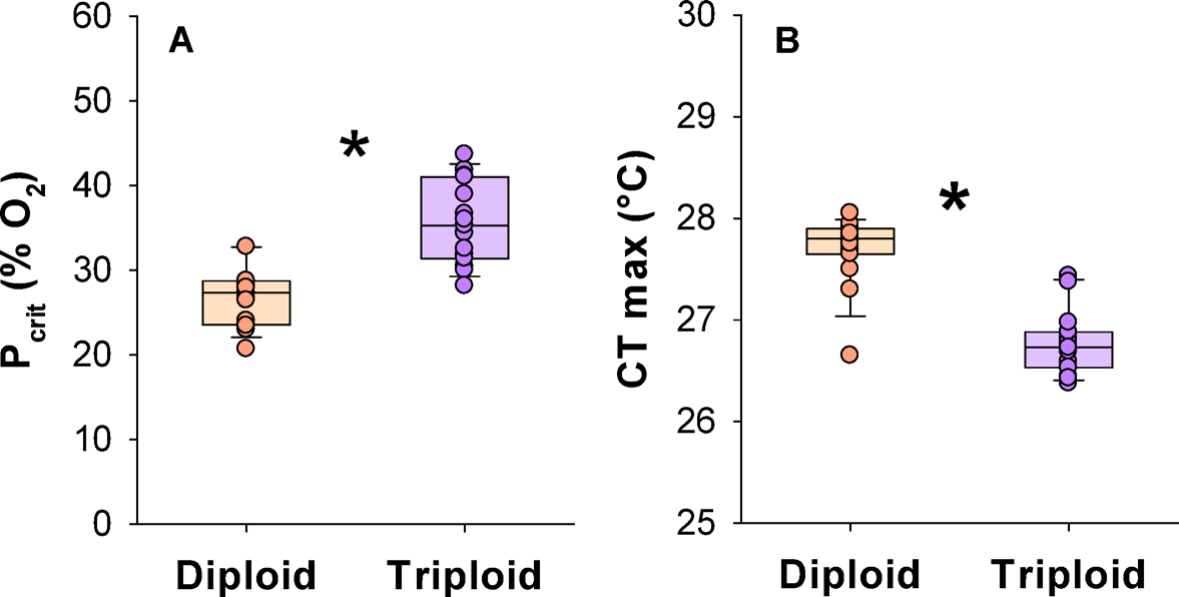
Environmental tolerance limits in larger diploid and triploid Atlantic salmon. The critical oxygen tension (P_crit_) (**A**) and the critical thermal maximum (CT max) (**B**). Data shown are boxplots together with individual data points. A significant difference is indicated with an asterisk (t-test, *P* < 0.05) (n = 15).

The P_crit_ was significantly lower in diploids being 26.9 ± 0.9%PO_2_ compared to 35.6 ± 1.3%PO_2_ in triploids (t-test, t = −5.647, DF = 28, *P* < 0.0001) (Fig. 3A), indicating poorer hypoxia tolerance in triploids. The CT max was significantly higher in diploids at 27.7 ± 0.09 °C compared to 26.8 ± 0.08 °C in triploids (t-test, t = 7.665, DF = 28, *P* < 0.001) (Fig. 3B), indicating poorer thermal tolerance in triploids.

**Fig. 4:**
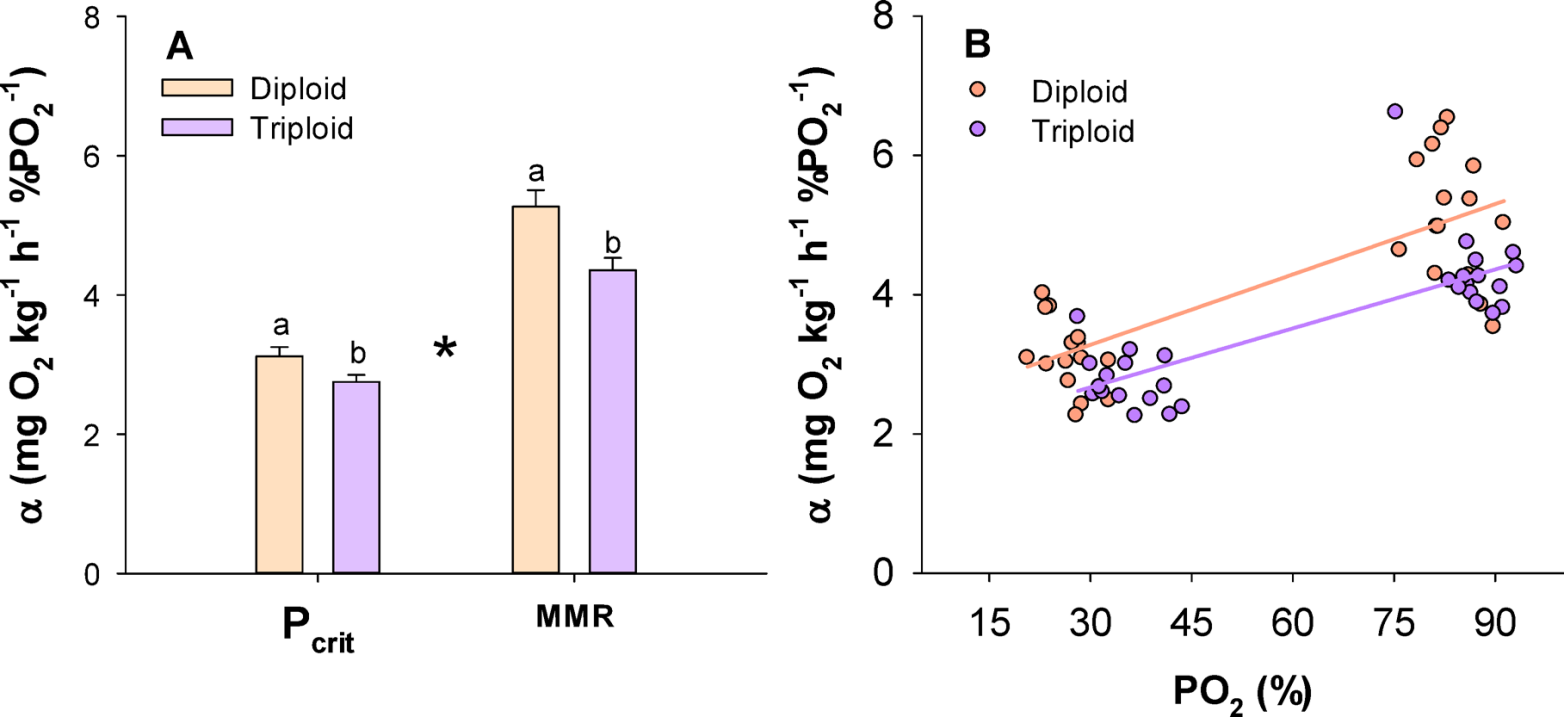
The oxygen extraction coefficient (α) at P_crit_ and MMR in larger diploid and triploid Atlantic salmon. Columns in (**A**) are mean ± s.e.m and the asterisk indicate a significant difference between metabolic rate traits while different letters indicate a significant difference between ploidies within a metabolic rate trait (two-way ANOVA and Hold-Sidak test, *P* < 0.05) (n = 15). Panel (**B**) depicts individual α values versus PO_2_, showing that α decrease in hypoxia and overall is lower in the triploid group.

In triploids the oxygen extraction coefficient, α, was significantly lower than in diploids (logged Two-way ANOVA, DF = 59, *P* = 0.001). Within traits, α was both lower in triploids at P_crit_ (Holm-Sidak test, t = 2.113 *P* = 0.039), and at MMR (Holm-Sidak test, t = 2.784, *P* = 0.007). Additionally, α values were overall lower at P_crit_ than at MMR regardless of ploidy and decreased at lower ambient oxygen levels (Holm-Sidak, t = 12.229, *P* < 0.001) (Fig. 4).

**Fig. 5:**
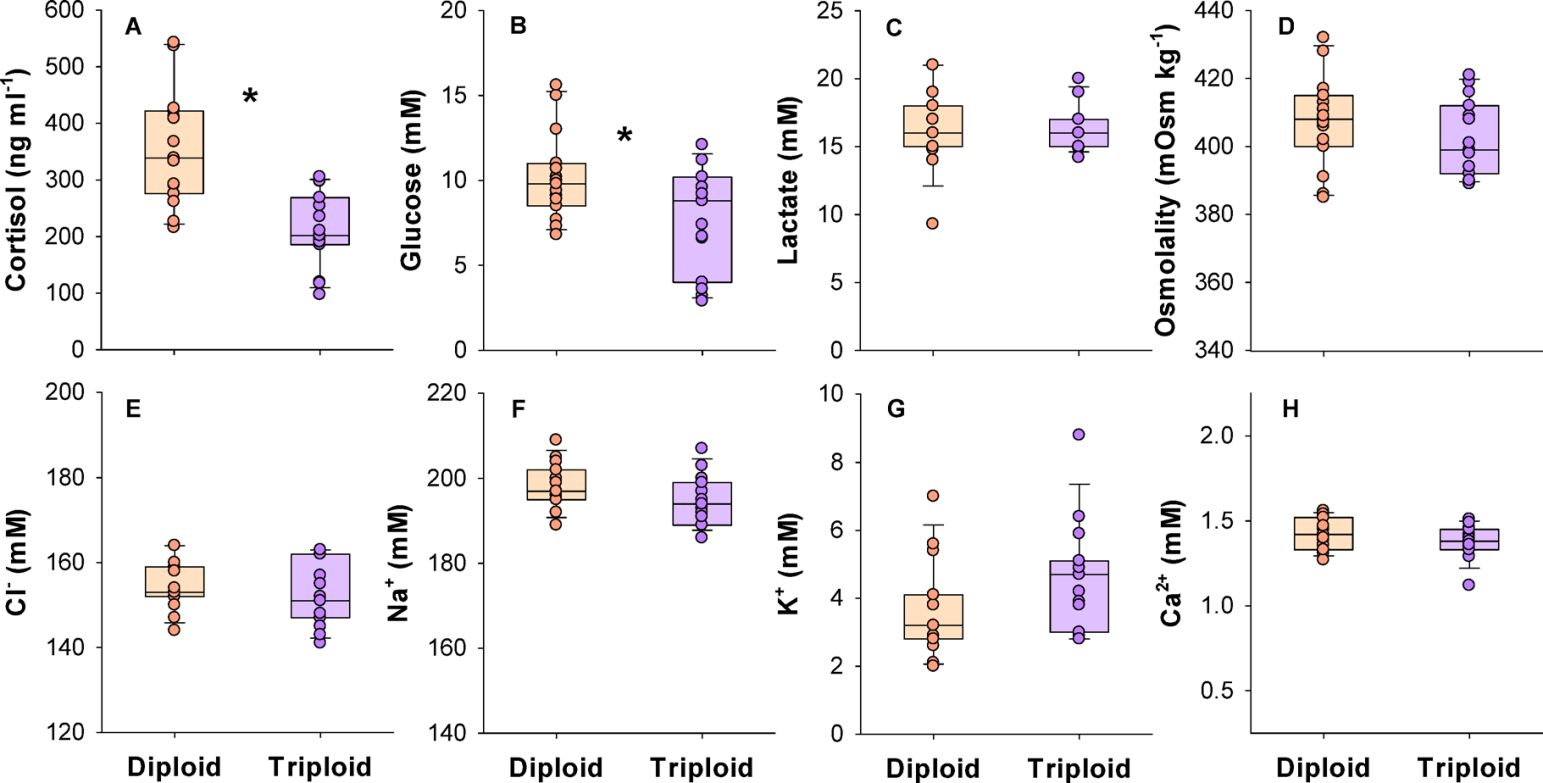
Haematological parameters in larger diploid and triploid Atlantic salmon following loss of equilibrium in the critical thermal maximum trials. Data shown as boxplots along with individual data points. A significant difference is indicated with an asterisk (t-test, *p* < 0.05) (n = 15).

The haematological parameters measured in fish immediately following loss of equilibrium imposed by acute thermal stress are summarized in Fig. 5. Plasma cortisol and glucose levels were both significantly higher in the diploids (t-test, DF = 28, t = 4.651, *P* < 0.001 for cortisol; t = 2.398, *p* = 0.0234 for glucose) (Fig. 5A and B). In the case of the other measured plasma parameters; lactate, osmolality, Cl^-^, Na^+^, K^+^, and Ca^2+^, the diploid and triploid groups were similar (t-test, DF = 28, *p *> 0.05) (Fig. 5C–H).

**Fig. 6:**
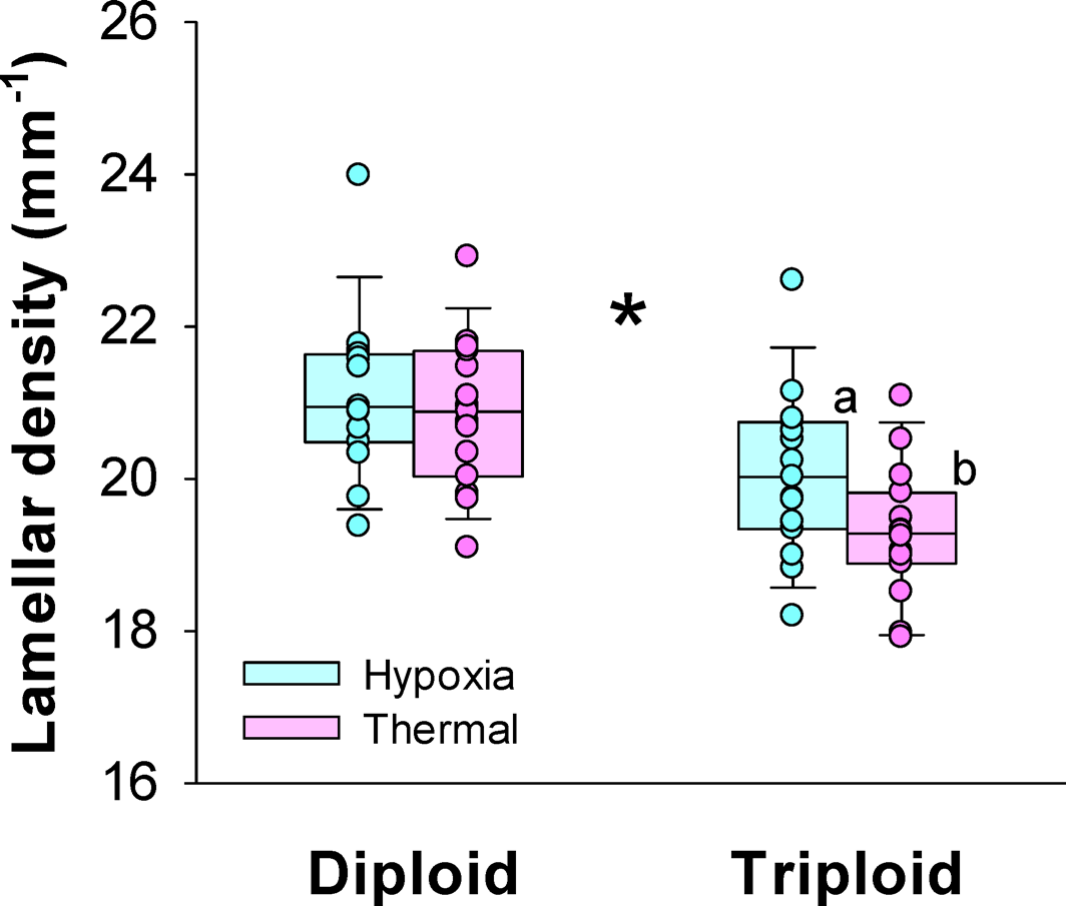
Lamellar density in larger diploid and triploid Atlantic salmon following exposure to hypoxia or thermal stress. Data shown as boxplots along with individual data points (n = 15). The asterisk indicates a significant difference between ploidies and different letters indicate a significant difference between trial type within a ploidy (two-way ANOVA and Hold-Sidak test, *P* < 0.05).

Histology revealed that the diploids had significantly higher lamellar densities than the triploids across trial type being 20.98 ± 0.18 mm^-1^ and 19.68 ± 0.19 mm^-1^, respectively, indicating higher gill surface areas in diploids (Two-way ANOVA, DF = 59, *P* < 0.001) (Fig. 6). Additionally, triploids subjected to hypoxia had higher lamellar densities than triploids subjected to thermal stress (Hold-Sidak test, t = 2.105, *P* = 0.040) (Fig. 6).

**Fig. 7:**
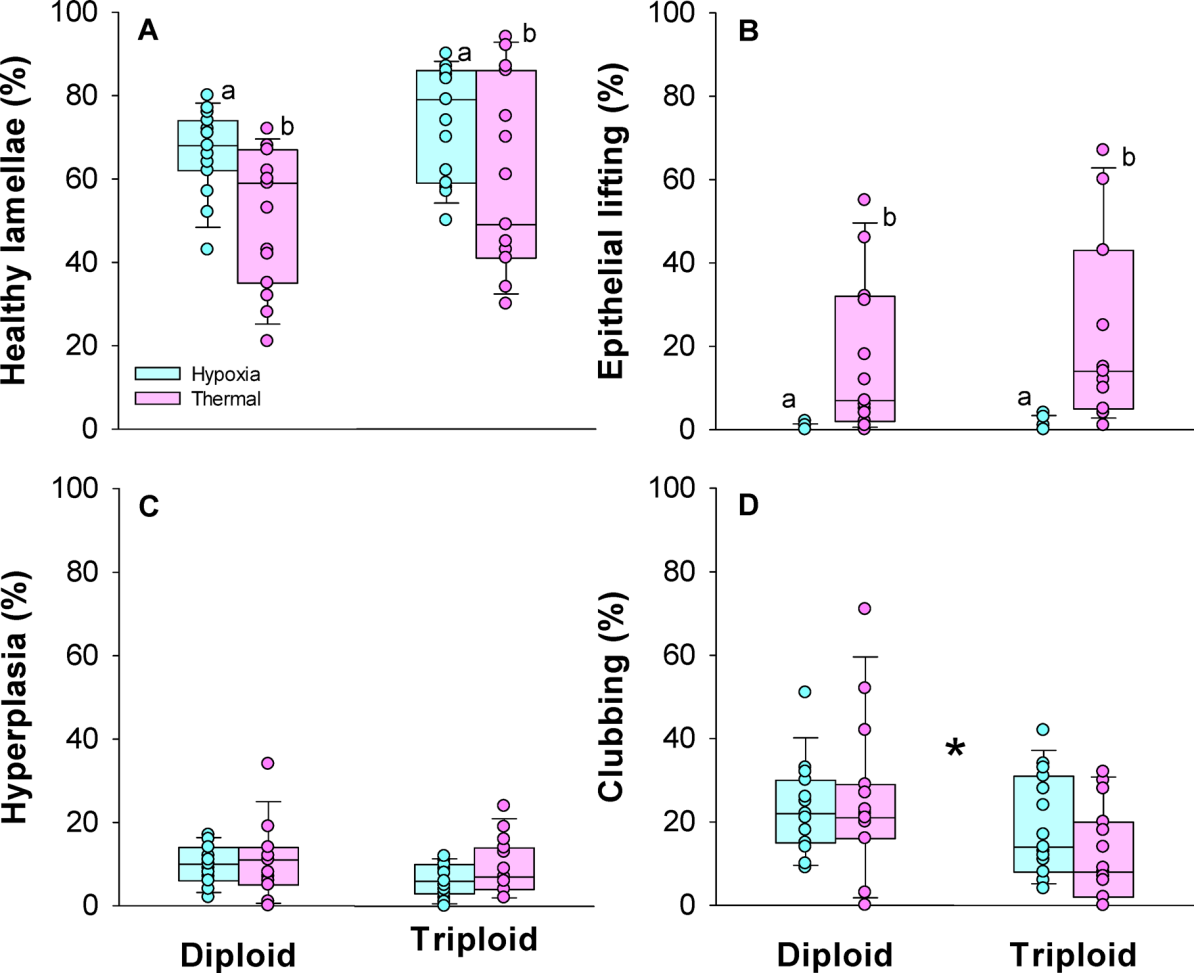
Gill histology health metrics in larger diploid and triploid Atlantic salmon following exposure to hypoxia or thermal stress. Data shown as boxplots along with individual data points (n = 15). An asterisk indicates a significant difference between ploidies and different letters indicate a significant difference between trial type within a ploidy (two-way ANOVA and Hold-Sidak test, *P* < 0.05).

The proportion of healthy gill lamellae did not differ between ploidies (Two-way ANOVA, DF = 59, *P* = 0.154) but differed significantly between the two trial types (*P* = 0.001). Specifically, the fish subjected to thermal stress had lower proportions of healthy lamellae within both ploidies compared to the fish subjected to hypoxia (Holm-Sidak test, t = 2.397, *P* = 0.020 in diploids, and t = 1.292, *P* = 0.016 in triploids) (Fig. 7A). A similar pattern was found for epithelial lifting where proportions were similar between diploids and triploids across trial type (Two-way ANOVA, DF = 59, P = 0.430), but differed between trial types where epithelial lifting frequently was observed in fish subjected to thermal stress and rarely observed in fish subjected to hypoxia (*P* < 0.001) (Fig. 7B). The proportion of hyperplasia was similar between both ploidy and trial type (Two-way ANOVA, DF = 59, *P* = 0.118 and *P* = 0.203, respectively) (Fig. 7C). Meanwhile, diploids were found to have a higher proportion of clubbing across trial type (two-way ANOVA, DF = 59, *P* = 0.022) (Fig. 7D). Additionally, moderate occurrences (< 10%) of lamellae with oedema were observed in 3 triploid fish and in 3 diploid fish. Finally, there were no observations of hyperemia, fusion, hypertrophy, aneurysm, and necrosis in any of the gill histology sections.

**Fig. 8:**
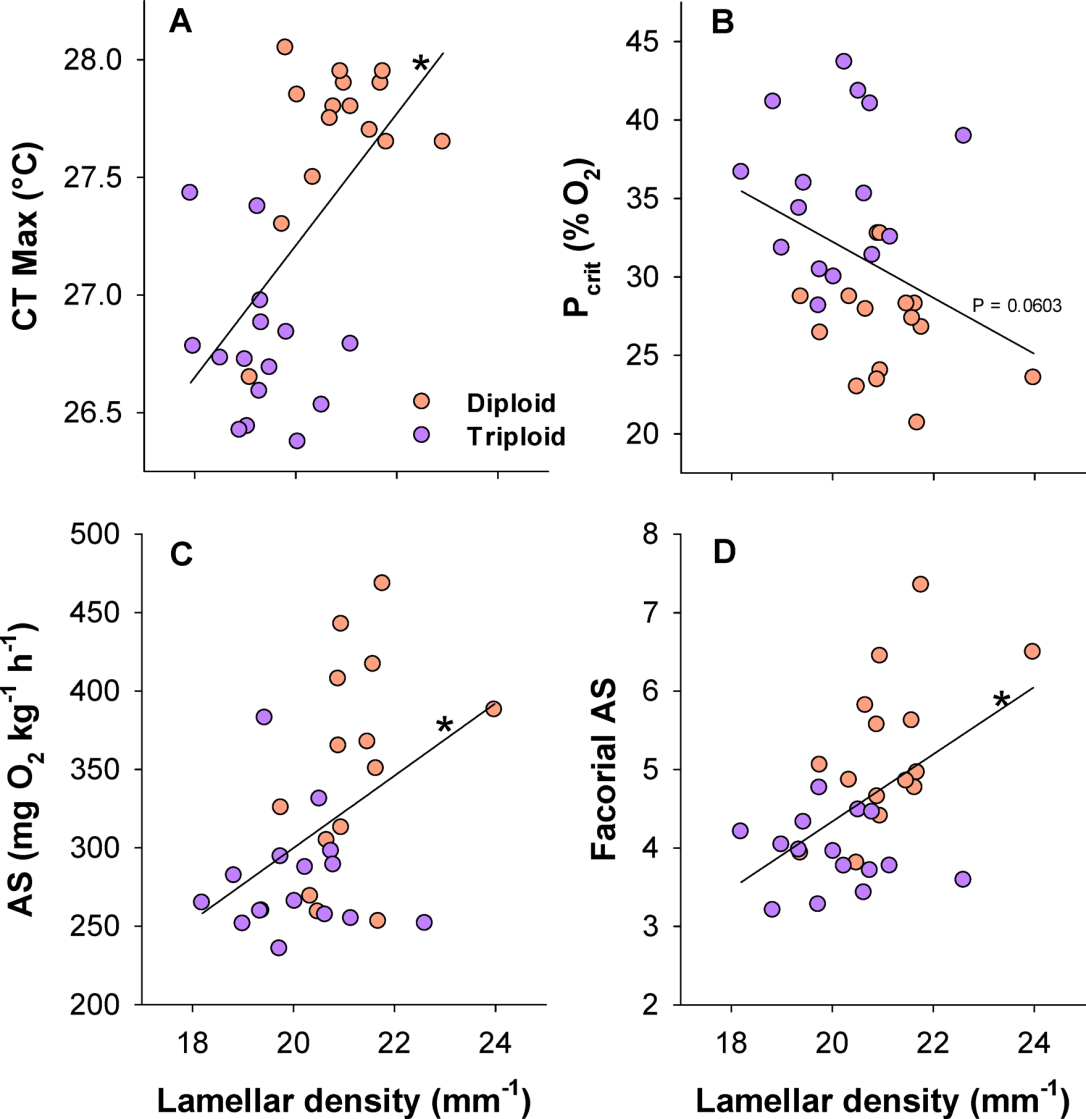
Correlations between physiological performance traits and lamellar density across larger diploid and triploid Atlantic salmon. (**A**) Critical thermal maximum (CT max), (**B**) The critical oxygen tension (P_crit_), (**C**): The aerobic scope (AS), and (**D**): The factorial AS. An asterisk indicates a significant correlation (Pearson’s correlation analyses, *P* < 0.05). Total n = 30 in each plot.

A higher lamellar density as a proxy of gill surface area was overall associated with improved physiological performance (Fig. 8). Specifically, Pearson’s correlation analyses showed that across groups the lamellar density was positively and significantly correlated with CT max (Coeff. = 0.593, *P* = 0.0005, N = 30), aerobic scope (Coeff. = 0.422, *P* = 0.0201, N = 30), and the factorial aerobic scope (Coeff. = 0.502, *P* = 0.00466, N = 30) (Fig. 8A, C and D), whereas the P_crit_ showed a negative correlation with lamellar density tending towards the significance threshold (Coeff. -0.347, *P* = 0.0603, N = 30) (Fig. 8B).

## Discussion

### Larger-sized triploid Atlantic salmon have diminished physiological efficiency

We hypothesized that larger-sized triploid Atlantic salmon would have reduced respiratory capacities and environmental tolerance limits when compared to similar-sized diploid counterparts. The experiments confirmed these predictions as the triploids were found to have higher SMR and lower MMR, resulting in lower aerobic scopes. Furthermore, triploids had worse hypoxia and thermal tolerances. These differences were theorised to be caused by physiological size-scaling effects together with larger cell sizes in triploids. This meant that being triploid should become a greater disadvantage at larger body sizes, particularly with regards to oxygen supply capacities. Indeed, the oxygen extraction coefficient, α, was found to be lower along ambient oxygen gradients. Impaired gill-oxygen supply was also implied in our histological assessments where the triploids were found to have lower lamellar densities, indicating lower gill surface areas. However, whether larger cell sizes in triploids contributed to reducing the functional surface areas of gill tissues was not directly discerned here.

Previous physiological studies on smaller-sized triploid and diploid Atlantic salmon have typically reported inconsistent or negligible differences^30,35^. By testing larger-sized fish, we hoped to demonstrate more obvious and consistent differences between triploid and diploid Atlantic salmon in accordance with our hypothesis. However, as can be seen on the figures, we still observed appreciable overlap between triploid and diploid individuals in the various physiological measurements. For instance, the P-value for MMR was close to the significance threshold. Meanwhile the P-values for environmental limits (P_crit_ and CT max) were highly significant (< 0.001). Some traits were therefore more obvious than others in their differences between ploidy groups. This also meant that the best performing triploids would have comparable performances to an average diploid. Still, we found that larger-sized triploid Atlantic salmon performed consistently worse across several metrics. It is therefore reasonable to conclude that they indeed are less physiologically robust compared to their diploid counterparts.

### Metabolic rates and oxygen supply limitation in larger triploid Atlantic salmon

A higher SMR in larger-sized triploid Atlantic salmon indicates higher basal maintenance costs when resting. On average the SMR was 17% higher in the triploids compared to the diploids in 12 °C full strength sea water. While this difference may seem minor, a 17% higher metabolic burden during routine conditions will accumulate to a substantial energetic deficit over time. Such deficits will have to be compensated by higher feed intake to obtain similar growth rates. In theory, this represents a disadvantage in aquaculture owing to reduced feed conversion rates but would also be a disadvantage in the wild where feeding opportunities are more limited.

Previous studies reported similar SMR between smaller-sized triploid and diploid Atlantic salmon tested across various acclimation temperatures spanning from 3 to 18 °C^30,35,61^. A similar SMR between juvenile triploid and diploid rainbow trout and brook trout have also been reported^33,34^, while on another occasion SMR was reported to be higher in juvenile triploid brook trout^28^. Additionally, juvenile triploid Atlantic salmon and brook charr were both found to have higher routine metabolic rates at lower acclimation temperatures while at higher acclimation temperatures routine metabolic rates were lower when compared to diploid counterparts^62^. As such, resting metabolic rates have previously mostly been found to be similar between smaller-sized triploid and diploid salmonids, although routine metabolic rates that encompass additional activities can differ across acclimation temperatures.

A potentially higher SMR in triploids may be ascribed to the increased cell maintenance costs from having 50% more DNA. However, this does not explain why SMR appear to be mostly similar among small juvenile triploid and diploid salmonids, while then becoming elevated in larger-sized triploids. An additional factor could perhaps be increased costs of osmoregulation in seawater for larger-sized triploid Atlantic salmon, while osmoregulatory costs presumably are lower for juvenile fish in freshwater.

The MMR was previously reported unaffected by ploidy in juvenile Atlantic salmon across different acclimation temperatures in freshwater^35^, while MMR was lower in seawater adapted triploid Atlantic salmon at 10.5 °C but not at 3 °C^30^. In the present study, MMR was lower in big triploids at 12 °C. Size, seawater, and higher temperatures may therefore all contribute to reduce the MMR in triploid Atlantic salmon relative to diploid counterparts. The underlying mechanism would likely be the lower surface to volume ratios impairing exchange rates across larger-sized cells while both increasing temperatures and higher salinities reduce dissolved oxygen levels in water.

Hypoxia tolerance expressed as the P_crit_ was substantially worse in big triploids in the present study. Reduced hypoxia tolerance in triploid salmonids, particularly at elevated temperatures, is one of the more consistent findings among previous studies^36–38^ and has also been reported in triploid zebrafish larvae^50^. For instance, triploid seawater adapted Atlantic salmon were observed to ram ventilate when being maintained in moderate hypoxia at 19 °C while diploids displayed normal gill ventilation^36^. Onset of ram ventilation was also observed at lower swimming speeds in triploid Atlantic salmon^30^. This points to a general issue with gill oxygen uptake capacity in triploids. In the present study, we found lower lamellar densities in triploids which indicate lower gill surface areas for gas exchange, providing a morphological link to impaired oxygen uptake capacity. Additionally, these experiments were performed at an optimal mid-temperature of 12 °C. The oxygen supply limitation observed in the larger triploid Atlantic salmon are therefore expected to be amplified further with increasing temperatures owing to metabolism inevitably increasing in ectothermic fish while less oxygen becomes available in the environment.

### Reduced thermal tolerance in larger triploid Atlantic salmon

The larger-sized triploid Atlantic salmon had a lower CT max than their diploid counterparts. Previously the CT max was found to be unaffected by ploidy in juvenile Atlantic salmon across different acclimation temperatures in freshwater^35^. Moreover, CT max was also similar between juvenile diploid and triploid brook trout and rainbow trout^34,63,64^.

Larger-sized fish tend to have lower thermal tolerance, and this may in part be explained by physiological scaling constraints causing oxygen supply limitation at increasing body sizes^40–42^. Owing to having larger-sized cells with lower surface to volume ratios, we therefore theorized that triploid fish would be subject to similar functional limitations as what will happen when fish becomes larger. That is, any size-driven impairment in physiological capacity should occur at relatively lower body sizes in triploids compared to diploids. Based on these considerations, the observed lower thermal tolerance in larger-sized triploid Atlantic salmon was therefore expected. This also align with lower aerobic scopes and oxygen extraction coefficients that were measured in the respirometry trials.

We also measured blood plasma parameters in fish subjected to the CT max trial to compare stress levels between ploidies at the point of loss of equilibrium. All the assessed plasma parameters resembled highly stressed fish and values of cortisol, glucose, lactate, and the major plasma ions were within similar ranges of seawater acclimated Atlantic salmon subjected to exhaustive exercise stress in critical swim speed trials^65,66^. As such, the CT max trial imposed high osmotic and ion disturbances as well as greatly elevated lactate values which signifies a substantial anaerobic load. The various plasma ions, osmolality and lactate levels were all similar between triploids and diploids at the point of loss of equilibrium. This suggests that both groups were experiencing the same amount of osmotic and anaerobic stress when they reached their respective CT max. The major difference being that the diploids were able to withstand higher temperatures before accumulating the same deficit in homeostasis that triggered loss of equilibrium. As such, lower thermal tolerance in larger triploid Atlantic salmon was indirectly associated with both reduced capacity for osmoregulation and oxygen uptake.

### Thermal stress caused alterations in gill morphology

The CT max test is the most common method to measure thermal tolerance in fish species^67^. While the endpoint of the test is loss of equilibrium, the method is generally not considered too severe as fish tend to recover quickly upon being returned to colder water. Furthermore, fish do not appear to suffer long-term consequences following CT max trials as evidenced from experiments with repeat testing and monitoring of growth rates^68,69^.

In the present study we assessed the gill histology of fish sampled following acute hypoxia exposure and following loss of equilibrium in a CT max trial. Interestingly, thermal stress appeared to cause histopathological changes that were not present in hypoxia exposed fish regardless of ploidy groups. This led us to wonder whether the CT max method could be more severe than previously assumed. Specifically, thermal stress resulted in a lower proportion of healthy lamellae, mainly owing to a high occurrence of epithelial lifting.

Epithelial lifting is a well-known histopathological response in the gills of fish species when exposed to various toxicants such as oils, ammonia, acids and metals^70,71^. The lifting of epithelial layers increases the intracellular spaces of secondary lamellae which then increases the diffusion distance between water and blood^72,73^. Furthermore, epithelial lifting is a rapid and reversible change and may therefore be considered an adaptive response to reduce uptake of pollutants from the environment^71,74^.

In the case of acute thermal stress, increasing the diffusion distance between the blood and the water via epithelial lifting could also be interpreted as a beneficial response. For instance, it may serve to slightly delay heating rates of circulating blood. Thermal stress in hyperosmotic seawater was here also associated with osmotic disturbances and highly elevated plasma sodium and chloride concentrations. Increased diffusion distances should initially have aided in preserving osmotic integrity by reducing ion leakage and passive water uptake prior to loss of equilibrium. On the other hand, increased diffusion distances would compromise gas transfer and eventually become detrimental as the fish struggle to supply enough oxygen, as indicated by high lactate levels in the diploid and triploid Atlantic salmon subjected to the CT max test. Nevertheless, epithelial lifting could serve as a useful response to balance the osmo-respiratory compromise of the gill during periods of thermal stress. In the future it would be interesting to investigate at what point epithelial lifting occur during a thermal challenge prior to physiological collapse and loss of equilibrium to further discern the role of this morphological response.

### Animal welfare in aquaculture and the future use of triploid Atlantic salmon

Norway is the world’s largest producer of farmed salmon. In 2023 the mortality rates in Norwegian salmon aquaculture were unfortunately the highest ever recorded where 63 million (16.7%) Atlantic salmon died during the seawater growth phase^75^. High mortality and poor animal welfare gives a negative reputation to the aquaculture industry, and there is presently a substantial political pressure to reduce mortalities. The ongoing moratorium on the use of triploid Atlantic salmon in sea cages in Norway therefore needs to be evaluated with this current situation in mind.

The welfare of triploid Atlantic salmon is mainly a concern during the latter marine growth phase, where mortalities are linked to delousing operations and other imposed handling stressors^13,14^. These field reports align with the results presented here where larger-sized triploid Atlantic salmon were physiologically compromised compared to their diploid counterparts. Lower aerobic scopes render triploids less flexible in coping with additional challenges in the farm environment such as stressors, parasites, or other prevailing health issues. For instance, infestation with the widespread salmon louse *Lepeophtheirus salmonis* may increase standard metabolic rates by ~ 25%^76^, leaving even less aerobic scope available when triploids become infested.

We also found that larger-sized triploids had reduced tolerance limits to hypoxia and thermal stress. Meanwhile, heatwaves and hypoxia events are both reoccurring issues in sea cage environments that are projected to become more frequent in the future owing to climate change^24,26,27,77^. In future aquaculture scenarios triploid Atlantic salmon will therefore become further disadvantaged.

Larger-sized triploid Atlantic salmon are still adequately functioning animals when maintained under fairly optimal conditions. However, rearing conditions cannot be expected to remain optimal at all times in industrial-scale marine aquaculture. The use of triploids will therefore impose a greater inherent risk of poor animal welfare. Considering that mortalities in Norwegian salmon aquaculture already are too high^75^, it becomes difficult to ethically justify the use of fish that are less physiologically robust. Meanwhile, the problem of escaped fish breeding in the wild will then remain unsolved for the time being. This imposes an ethical dilemma between animal welfare and conservation of wild populations. Emerging biotechnologies may eventually provide alternative solutions to produce sterile diploid salmon that do not suffer impaired robustness^78^

## Data Availability

Raw data is available upon request to the corresponding author.

## References

[1] Teletchea, F. & Fontaine, P. Levels of domestication in fish: Implications for the sustainable future of aquaculture. *Fish Fish.* **15**, 181–195 (2014).

[2] Glover, K. A. et al. Half a century of genetic interaction between farmed and wild Atlantic salmon: Status of knowledge and unanswered questions. *Fish Fish.* **18**, 890–927 (2017).

[3] Gjerde, B. Response to individual selection for age at sexual maturity in Atlantic salmon. *Aquaculture* **38**, 229–240 (1984).

[4] Gjedrem, T., Gjoen, H. M. & Gjerde, B. Genetic origin of Norwegian farmed Atlantic salmon. *Aquaculture* **98**, 41–50 (1991).

[5] Solberg, M. F., Robertsen, G., Sundt-Hansen, L. E., Hindar, K. & Glover, K. A. Domestication leads to increased predation susceptibility. *Sci. Rep.* **10**, 1929 (2020). 32029847 10.1038/s41598-020-58661-9 PMC7005312

[6] Bolstad, G. H. et al. Introgression from farmed escapees affects the full life cycle of wild Atlantic salmon. *Sci Adv* **7**, abj3397 (2021). 10.1126/sciadv.abj3397 PMC8694624 34936452

[7] Besnier, F. et al. Introgression of domesticated salmon changes life history and phenology of a wild salmon population. *Evol. Appl.* **15**, 853–864 (2022). 35603027 10.1111/eva.13375 PMC9108307

[8] Vollset, K. W. et al. Wild salmonids are running the gauntlet of pathogens and climate as fish farms expand northwards. *ICES J. Mar. Sci.* **78**, 388–401 (2021).

[9] Johnsen, I. A. et al. Salmon lice-induced mortality of Atlantic salmon during post-smolt migration in Norway. *ICES J. Mar. Sci.* **78**, 142–154 (2020).

[10] Benfey, T. J. Effectiveness of triploidy as a management tool for reproductive containment of farmed fish: Atlantic salmon (*Salmo salar*) as a case study. *Rev. Aquac.* **8**, 264–282 (2016).

[11] Piferrer, F. et al. Polyploid fish and shellfish: Production, biology and applications to aquaculture for performance improvement and genetic containment. *Aquaculture* **293**, 125–156 (2009).

[12] Small, S. A. & Benfey, T. J. Cell-size in triploid salmon. *J. Exp. Zool.* **241**, 339–342 (1987).

[13] Madaro, A., Kjøglum, S., Hansen, T., Fjelldal, P. G. & Stien, L. H. A comparison of triploid and diploid Atlantic salmon (*Salmo salar*) performance and welfare under commercial farming conditions in Norway. *J. Appl. Aquac.* **34**(4), 1021–1035 (2022).

[14] Stien, L. H., Thompson, C., Fjelldal P. G., Oppedal, F., Kristiansen, T. S., Sæther, P. A., Bølgen, P. M. & Martinsen, L. Production, fasting and delousing of triploid and diploid salmon in Northern Norway - Report for the 2020-generation. Bergen (2023).

[15] Kyst (2021). URL: https://www.kyst.no/mattilsynet-nrs-triploid/avslutter-produksjon-av-triploid-laks/143561) (In Norwegian) (accessed 02.11.25).

[16] Stien, L. H., Sæther, P. A., Kristiansen, T. S., Fjelldal, P. G. & Sambraus, F. First collective report: welfare of triploid salmon in northern Norway from transfer till slaughter, 2014–2017 transfers., Rapport fra Havforskningen (2019).

[17] Aunsmo, A. et al. Triploid Atlantic salmon (*Salmo salar*) may have increased risk of primary field outbreaks of infectious salmon anaemia. *J. Fish. Dis.* **45**, 1733–1743 (2022). 35914108 10.1111/jfd.13695 PMC9805046

[18] Fraser, T. W. K. et al. The effect of triploidy on the culture performance, deformity prevalence, and heart morphology in Atlantic salmon. *Aquaculture* **416**, 255–264 (2013).

[19] Taylor, J. F. et al. Ploidy and family effects on Atlantic salmon (Salmo salar) growth, deformity and harvest quality during a full commercial production cycle. *Aquaculture* **410**, 41–50 (2013).

[20] Taylor, J. F. et al. Triploid Atlantic salmon growth is negatively affected by communal ploidy rearing during seawater grow-out in tanks. *Aquaculture* **432**, 163–174 (2014).

[21] Crouse, C., Davidson, J., May, T., Summerfelt, S. & Good, C. Production of market-size European strain Atlantic salmon (*Salmo**salar*) in land-based freshwater closed containment aquaculture systems. *Aquacult. Eng.* **92**, 102138 (2021).

[22] Fraser, T. W. K., Fjelldal, P. G., Hansen, T. & Mayer, I. Welfare considerations of triploid fish. *Rev. Fish. Sci.* **20**, 192–211 (2012).

[23] Rimstad, E., Basic, D., Espmark, Å.M.O., Fraser, T., Gulla, S., Johansen, J., Mo, T.A., Olesen, I., Olsen, R.E. Triploid Atlantic salmon in aquaculture: Consequences for fish health and welfare under farming conditions. Scientific opinion of the panel on animal health and welfare. VKM report 2023:22, ISBN: 978–82–8259–433–2, ISSN: 2535–4019. Norwegian scientific committee for food and environment (VKM), Oslo, Norway (2023).

[24] Solstorm, D. et al. Dissolved oxygen variability in a commercial sea-cage exposes farmed Atlantic salmon to growth limiting conditions. *Aquaculture* **486**, 122–129 (2018).

[25] Oldham, T., Oppedal, F. & Dempster, T. Cage size affects dissolved oxygen distribution in salmon aquaculture. *Aquac. Environ. Interact.* **10**, 149–156 (2018).

[26] Wade, N. M. et al. Effects of an unprecedented summer heatwave on the growth performance, flesh colour and plasma biochemistry of marine cage-farmed Atlantic salmon (*Salmo salar*). *J. Therm. Biol.* **80**, 64–74 (2019). 30784489 10.1016/j.jtherbio.2018.12.021

[27] Meng, H., Hayashida, H., Norazmi-Lokman, N. H. & Strutton, P. G. Benefits and detrimental effects of ocean warming for Tasmanian salmon aquaculture. *Cont. Shelf Res.* **246**, 104829 (2022).

[28] O’Donnell, K. M., Macrae, K. L., Verhille, C. E., Charles, F. D. & Benfey, T. Standard metabolic rate of juvenile triploid brook charr *Salvelinus**fontinalis*. *Aquaculture* **479**, 85–90 (2017).

[29] Bernier, N. J., Brauner, C. J., Healt, J. W. & Randall, D. J. Oxygen and carbon dioxide transport during sustained exercise in diploid and triploid chinook salmon (*Oncorhynchus tshawytscha*). *Can. J. Fish. Aquat. Sci.* **6**(19), 1797–1805 (2004).

[30] Riseth, E. N., Fraser, T. W. K., Sambraus, F., Stien, L. H. & Hvas, M. Is it advantageous for Atlantic salmon to be triploid at lower temperatures?. *J. Therm. Biol* **89**, 102548 (2020). 32364990 10.1016/j.jtherbio.2020.102548

[31] Small, S. A. & Randall, D. J. Effects of triploidy on the swimming performance of coho salmon (*Oncorhynchus kitsch*). *Can. J. Fish. Aquat. Sci.* **46**, 243–245 (1989).

[32] Stillwell, E. J. & Benfey, T. J. The critical swimming velocity of diploid and triploid brook trout. *J. Fish. Biol.* **51**, 650–653 (1997).

[33] Hyndman, C. A., Kieffer, J. D. & Benfey, T. J. The physiological response of diploid and triploid brook trout to exhaustive exercise. *Comp. Biochem. Physiol. A* **134**(1), 167–179 (2003). 10.1016/s1095-6433(02)00245-3 12507620

[34] Scott, M. A., Dhillon, R. S., Schulte, P. M. & Richards, J. G. Physiology and performance of wild and domestic strains of diploid and triploid rainbow trout (*Oncorhynchus**mykiss*) in response to environmental challenges. *Can. J. Fish. Aquat. Sci.* **72**, 125–134 (2015).

[35] Bowden, A. J., Andrewartha, S. J., Elliott, P. B., Frappell, N. G. & Clark, T. D. Negligible differences in metabolism and thermal tolerance between diploid and triploid Atlantic salmon (*Salmo**salar* L.). *J. Exp. Biol.* **221**, 1–9 (2018). 10.1242/jeb.166975 29361579

[36] Hansen, T. J. et al. Effect of water oxygen level on performance of diploid and triploid Atlantic salmon post- smolts reared at high temperature. *Aquaculture* **435**(1), 354–360 (2015).

[37] Benfey, T. J. & Devlin, R. H. Ploidy has minimal effect on hypoxia tolerance at high temperature in rainbow trout (*Oncorhynchus mykiss*). *Physiol. Biochem. Zool.* **91**, 1091–1101 (2018). 30285539 10.1086/700218

[38] Jensen, R. R. & Benfey, T. J. Acclimation to warmer temperature reversibly improves high-temperature hypoxia tolerance in both diploid and triploid brook charr, *Salvelinus fontinalis*. *Compar Biochem Physiol A* **264**, 111099 (2022). 10.1016/j.cbpa.2021.111099 34718146

[39] Verhille, C., Anttila, K. & Farrell, A. P. A heart to heart on temperature: impaired temperature tolerance of triploid rainbow trout (*Oncorhynchus mykiss*) due to early onset of cardiac arrhythmia. *Compar Biochem Physiol A* **164**, 653–657 (2013). 10.1016/j.cbpa.2013.01.011 23370292

[40] Pörtner, H. O. & Farrell, A. P. Physiology and climate change. *Science* **322**, 690–692 (2008). 18974339 10.1126/science.1163156

[41] Clark, T. D. et al. Physiological benefits of being small in a changing world: Responses of Coho salmon (*Oncorhynchus kisutch*) to an acute thermal challenge and a simulated capture event. *PLoS ONE* **7**, e39079 (2012). 22720035 10.1371/journal.pone.0039079 PMC3374769

[42] Pauly, D. & Cheung, W. W. L. Sound physiological knowledge and principles in modelling shrinking of fishes under climate change. *Glob Change Biol* **24**, e15–e26 (2018). 10.1111/gcb.13831 28833977

[43] Daufresne, M., Lengfellner, K. & Sommer, U. Global warming benefits the small in aquatic ecosystems. *Proc. Natl. Acad. Sci. USA* **106**(31), 12788–12793 (2009). 19620720 10.1073/pnas.0902080106 PMC2722360

[44] Gardner, J. L., Peters, A., Kearney, M. R., Joseph, L. & Heinsohn, R. Declining body size: A third universal response to warming?. *Trends Ecol. Evol.* **26**(6), 285–291 (2011). 21470708 10.1016/j.tree.2011.03.005

[45] Sheridan, J. A. & Bickford, D. Shrinking body size as an ecological response to climate change. *Nat. Clim. Chang.* **1**, 401–406 (2011).

[46] van de Pol, I. L. E., Wilco, G. F. & Verberk, C. E. P. Triploidy in zebrafish larvae: Effects on gene expression, cell size and cell number, growth development and swimming performance. *PLoS ONE* **15**(3), e0229468 (2020). 32119699 10.1371/journal.pone.0229468 PMC7051096

[47] Weatherley, A. H., Gill, H. S. & Lobo, A. F. Recruitment and maximal diameter of axial muscle fibres in teleosts and their relationship to somatic growth and ultimate size. *J. Fish Biol.* **33**, 851–859 (1988).

[48] Johnsen, C. A. et al. Seasonal changes in muscle structure and flesh quality of 0+ and 1+ Atlantic salmon (*Salmo**salar* L.): Impact of feeding regime and possible roles of ghrelin. *Aquac Nutr* **19**, 15–34 (2013).

[49] Johnston, I. A., Strugnell, G., McCracken, M. L. & Johnstone, R. Muscle growth and development in normal-sex-ratio and all-female diploid and triploid Atlantic salmon. *J. Exp. Biol.* **202**(15), 1991–2016 (1999). 10393816 10.1242/jeb.202.15.1991

[50] Hermaniuk, A., van de Pol, I. L. E. & Verberk, W. C. E. P. Are acute and acclimated thermal effects on metabolic rate modulated by cell size? A comparison between diploid and triploid zebrafish larvae. *J Exp Biol* **224**, 227124 (2021). 10.1242/jeb.227124 33257437

[51] van de Pol, I. L. E., Hermaniuk, A. & Verberk, W. C. E. P. Interacting effects of cell size and temperature on gene expression, growth, development and swimming performance in larval zebrafish. *Front. Physiol.* **12**, 738804 (2021). 34950046 10.3389/fphys.2021.738804 PMC8691434

[52] Chabot, D., Steffensen, J. F. & Farrell, A. P. The determination of standard metabolic rate in fishes. *J Fish Biol* **88**, 81–121 (2016). 26768973 10.1111/jfb.12845

[53] Benfey, T. J., Sutterlin, A. M. & Thompson, R. J. Use of erythrocyte measurements to identify triploid salmonids. *Can. J. Fish. Aquat. Sci.* **41**, 980–984 (1984).

[54] Mitchell, S. O., Scholz, F., Marcos, M. & Rodger, H. Sampling artefacts in gill histology of freshwater Atlantic salmon (*Salmo salar*). *Bull Eur Assoc Fish Pathol* **43**(1), 1–11 (2023).

[55] Wolf, J. C. et al. Nonlesions, misdiagnoses, missed diagnoses, and other interpretive challenges in fish histopathology studies: A guide for investigators, authors, reviewers, and readers. *Toxicol. Pathol.* **43**(3), 297–325 (2015). 25112278 10.1177/0192623314540229

[56] Clark, T. D., Sandblom, E. & Jutfelt, F. Aerobic scope measurements of fishes in an era of climate change: Respirometry, relevance and recommendations. *J. Exp. Biol.* **216**, 2771–2782 (2013). 23842625 10.1242/jeb.084251

[57] Hvas, M. & Oppedal, F. Influence of experimental set-up and methodology for measurements of metabolic rates and critical swimming speed in Atlantic salmon *Salmo salar*. *J. Fish Biol.* **95**, 893–902 (2019). 31265133 10.1111/jfb.14087

[58] Ern, R., Norin, T., Gamperl, A. K. & Esbaugh, A. J. Oxygen dependence of upper thermal limits in fishes. *J. Exp. Biol.* **219**, 3376–3383 (2016). 27591316 10.1242/jeb.143495

[59] Reemeyer, J. E. & Rees, B. B. Standardizing the determination and interpretation of Pcrit in fishes. *J Exp Biol* **222**, jeb210633 (2019). 31511343 10.1242/jeb.210633

[60] Seibel, B. A. et al. Oxygen supply capacity breathes new life into the critical oxygen partial pressure (P_crit_). *J Exp Biol* **224**, jeb242210 (2021). 33692079 10.1242/jeb.242210

[61] Lijalad, M. & Powell, M. D. Effects of lower jaw deformity on swimming performance and recovery from exhaustive exercise in triploid and diploid Atlantic salmon *Salmo salar* L. *Aquaculture* **290**, 145–154 (2009). 10.3354/dao02056 19593934

[62] Atkins, M. E. & Benfey, T. J. Effect of acclimation temperature on routine metabolic rate in triploid salmonids. *Comp Biochem Physiol A* **149**, 157–161 (2008). 10.1016/j.cbpa.2007.11.004 18155947

[63] Benfey, T. J. et al. Critical thermal maxima of diploid and triploid brook charr *Salvelinus**fontinalis*. *Environ. Biol. Fish.* **49**, 259–264 (1997).

[64] Galbreath, P. F., Adams, N. D., Sherrill, L. W. & Martin, T. H. Thermal tolerance of diploid versus triploid rainbow trout and brook trout assessed by time to chronic lethal maximum. *Environ. Biol. Fish.* **75**, 183–193 (2006).

[65] Hvas, M., Nilsen, T. O. & Oppedal, F. Oxygen uptake and osmotic balance of Atlantic salmon in relation to exercise and salinity acclimation. *Front Mar Sci* **5**, 368 (2018).

[66] Hvas, M., Stien, L. H. & Oppedal, F. The effect of fasting period on swimming performance, blood parameters and stress recovery in Atlantic salmon post smolts. *Comp. Biochem. Physiol. A* **255**, 110913 (2020). 10.1016/j.cbpa.2021.110913 33524618

[67] Desforges, J. E. et al. The ecological relevance of critical thermal maxima methodology for fishes. *J Fish Biol* **102**, 1000–1016 (2023). 36880500 10.1111/jfb.15368

[68] Morgan, R., Finnøen, F. & Jutfelt, F. CTmax is repeatable and doesn’t reduce growth in zebrafish. *Sci. Rep.* **8**, 7099 (2018). 29740113 10.1038/s41598-018-25593-4 PMC5940882

[69] O’Donnell, M. J., Regish, A. M., McCormick, S. D. & Letcher, B. H. How repeatable is CTmax within individual brook trout over short- and long-time intervals?. *J. Therm. Biol* **89**, 102559 (2020). 32364992 10.1016/j.jtherbio.2020.102559

[70] Pandey, S. et al. Effects of exposure to multiple trace metals on biochemical, histological and ultra-structural features of gills of a freshwater fish *Channa**punctata**Bloch*. *Chem. Biol. Interact.* **174**(3), 183–192 (2008). 18586230 10.1016/j.cbi.2008.05.014

[71] Nascimento, A. A., Araujo, F. G., Gomes, I. D., Mendes, R. M. & Sales, A. Fish gills alterations as potential biomarkers of environmental quality in a eutrophized tropical river in south-eastern Brazil. *Anat. Histol. Embryol.* **41**, 209–216 (2012). 22211803 10.1111/j.1439-0264.2011.01125.x

[72] Simonato, J. D., Guedes, C. L. B. & Martinez, C. B. R. Biochemical, physiological, and histological changes in the neotropical fish *Prochilodus linetus* exposed to diesel oil. *Ecotoxicol. Environ. Saf.* **69**, 112–120 (2008). 17368761 10.1016/j.ecoenv.2007.01.012

[73] Elshaer, F., Allah, H. & Bakry, S. Histopathological alterations in gills of some poecilid fishes after exposure to bisphenol-a. *World J Fish Mar Sci* **5**(6), 693–700 (2015).

[74] Rašković, B. et al. Histopathological indicators: a useful fish health monitoring tool in common carp (*Cyprinus carpio* Linnaeus, 1758) culture. *Cent. Eur. J. Biol.* **8**(10), 975–985 (2013).

[75] Sommerset et al. Fiskehelserapporten 2023, Veterinærinstituttets rapportserie nr. 8a/2024 (2024).

[76] Hvas, M. & Bui, S. Energetic costs of ectoparasite infection in Atlantic salmon. *J Exp Biol* **225**, jeb243300 (2022). 34931653 10.1242/jeb.243300

[77] Stehfest, K. M., Carter, C. G., McAllister, J. D., Ross, J. D. & Semmens, J. M. Response of Atlantic salmon *Salmo salar* to temperature and dissolved oxygen extremes established using animal-borne environmental sensors. *Sci. Rep.* **7**, 4545 (2017). 28674437 10.1038/s41598-017-04806-2 PMC5495760

[78] Kleppe, L. et al. Full production cycle performance of gene-edited, sterile Atlantic salmon: growth, smoltification, welfare indicators and fillet composition. *Aquaculture* **560**, 738456 (2022).

